# Recent Progress of Artificial Intelligence Application in Polymer Materials

**DOI:** 10.3390/polym17121667

**Published:** 2025-06-16

**Authors:** Teng Long, Qianqian Pang, Yanyan Deng, Xiteng Pang, Yixuan Zhang, Rui Yang, Chuanjian Zhou

**Affiliations:** 1 School of Materials Science & Engineering, Shandong University, Jinan 250061, China; 2 State Key Laboratory of Coatings for Advanced Equipment, Jinan 250061, China; 3 School of Information Science and Engineering, Zaozhuang University, Zaozhuang 277160, China; 4 Institute of Materials Science, Technical University of Darmstadt, 64287 Darmstadt, Germany

**Keywords:** artificial intelligence, polymer materials, database, descriptors, algorithm

## Abstract

Artificial intelligence (AI) technology has made remarkable progress in polymer materials, which has changed polymer science significantly. However, this community still relies heavily on the traditional research paradigm instead of the data-driven paradigm. This review advocates for a fundamental paradigm shift in polymer research from traditional experience-driven methods to data-driven approaches enabled by AI. While AI has made transformative advances in polymer design, property prediction, and process optimization, the field remains anchored in conventional methodologies. AI’s computational advantages against persistent barriers are also evaluated, such as data scarcity, inadequate material descriptors, and algorithmic complexity. Potential solutions, including collaborative data platforms, domain-adapted descriptor frameworks, and active learning strategies, are also discussed. Furthermore, we demonstrate how high-quality data and explainable AI methodologies overcome computational limitations while ensuring result credibility in other areas, which can benefit polymer research. Ultimately, this work provides a roadmap for accelerating the sustainable convergence of data-driven AI innovation with polymer science.

## 1. Introduction

Over the past century, polymer materials have undergone rapid development and have become indispensable components in modern society. Their application domains have continuously expanded to encompass key industries such as energy [1], healthcare [2,3], biomedical [4], electronics [5,6,7], and construction [8]. Although the production volumes of commodity polymers such as polyethylene, polypropylene, and polystyrene have surpassed those of traditional metallic materials like steel [9], these polymers still exhibit limitations in terms of mechanical properties and thermal stability, which constrain their application. To overcome existing performance bottlenecks of polymer materials, the design and fabrication of novel high-performance polymers have emerged as a critical research frontier in materials science [10]. Nevertheless, the development workflow for polymer materials is complex, involving multiple interrelated stages, including molecular structure design, controllable synthetic characterization, and scalable process optimization [11,12]. This journey typically spans more than a decade and requires substantial research and development investment. Conventional research approaches heavily rely on experience-driven trial-and-error methods, which are inefficient when navigating through the high-dimensional and nonlinear chemical space, thereby significantly limiting the speed and efficiency of new material discovery. Consequently, there is an urgent need for more efficient research paradigms to accelerate new polymer materials development [13].

**Figure 1:**
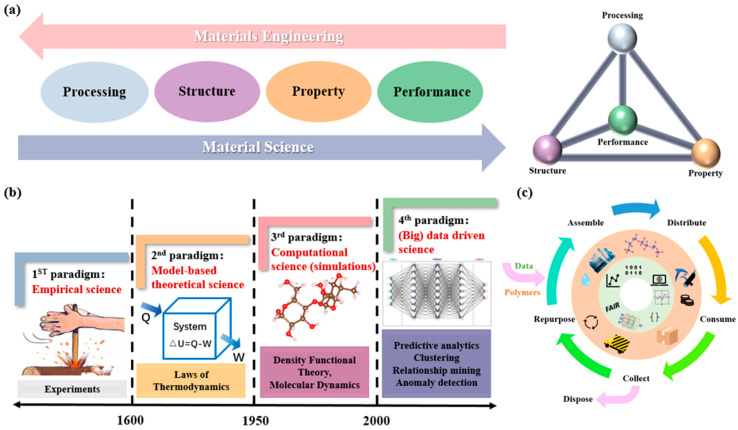
Multi-paradigm development and data-driven process of materials science and engineering. (**a**) Schematic diagram of PSPP relationship; (**b**) four major paradigms of materials development; (**c**) life cycle and data of polymer materials. This figure is inspired by works from Deagen et al. [38].

In recent years, the rapid development of artificial intelligence (AI) technologies has significantly prompted the advancement of materials science [14,15]. This subject focuses on the understanding of the relationships among processing, structure, properties, and performance (PSPP) [16] of different materials (Figure 1). Currently, such study is mainly conducted by experiment paradigms, which is relative slow and inefficient. By integrating AI into this framework, these multidimensional relationships can be explored in a more efficient and fast way, thereby accelerating materials research. Related works are now widely applied in energy materials [17,18,19], mechanical materials [20,21], bionic materials [22,23,24], medical materials [25], and other fields [26,27,28]. Their applications involve the composition optimization [29], experiment cost deduction [30], properties prediction (including Young’s modulus [31], melting temperature [32,33], thermal stability, and thermal conductivity [34,35]), and characterization analysis (such as powder diffraction (PD), pair distribution function (PDF), small-angle scattering (SAS), inelastic neutron scattering (INS), and X-ray absorption spectroscopy (XAS) [36,37]). These applications not only improve research efficiency, but also provide strong support for the design and development of new materials.

Compared to inorganic material systems such as metals and ceramics, polymer materials exhibit significant complexity and diversity in their microstructures [39]. Typical polymers exhibit the flexibility of molecular chains, compositional polydispersity, sequence randomness, hierarchical multi-level structures, and strong coupling between processing conditions and final properties. On the one hand, these features endow polymers with excellent mechanical, thermal, electrical, and processing performance [40]; on the other hand, they substantially increase the dimensionality of design variables and the complexity of modeling, rendering traditional “trial-and-error” approaches inadequate for precise design [41]. The emergence of AI, with strong generalization and feature extraction capabilities, has established a new paradigm for the structural design, property prediction, and process optimization of polymer materials [42]. AI can efficiently identify latent relationships within high-dimensional, nonlinear, and multivariable PSPP spaces, thereby enabling coordinated design across multiple structural levels of polymers and significantly enhancing the efficiency and success rate of materials development.

As a result, extensive research efforts have been devoted to study the relationship between materials structure and properties, and the relationship between material performance and processing conditions by AI [43]. For instance, deep neural networks (DNNs) [44] and graph neural networks (GNNs) [45] have been employed to construct predictive models that map molecular structures to properties such as glass transition temperature, modulus, and thermal stability. Reinforcement learning (RL) [46] algorithms have also been applied to automatically optimize polymerization process parameters. These advances collectively demonstrate the unique strengths of AI in uncovering hidden patterns and accelerating design in complex polymer systems. Nevertheless, the successful application of AI in polymer science still faces several critical challenges [47]. First, high-quality and diverse datasets are not available, yet the acquisition of such data is high-cost and low-efficiency [48]. Second, descriptors are not effective due to the multi-scale and multidimensional structural features of polymers [49]. Third, the lack of interpretability of the results limits researchers’ ability to understand the underlying scientific relationships [50]. Therefore, reducing dependence on large-scale labeled datasets, establishing effective descriptors, and improving model interpretability represent potential strategies to overcome these challenges.

Thus, this paper will first introduce the current applications of AI in polymer science, with a focus on its practical roles in polymer design, property prediction, and process optimization. Next, we will analyze the key challenges at the intersection of AI and polymer materials—namely, difficulties in data acquisition, insufficient polymer description, and poor interpretability—and the corresponding solutions. Finally, the future of this area will be discussed.

## 2. The Application of AI in Polymer Materials

AI techniques are revolutionizing polymer materials science by enabling predictions and optimizations. In order to discuss the recent progress, this section will discuss this topic from three different aspects: the development of AI technique, the application of AI in property/structure relationship research, and the application in processing-performance research.

### 2.1. Foundations and Applications of Machine Learning in Materials Science

By emulating the behavior of human neural networks (NNs), ML identifies and learns patterns within data, thereby establishing complex nonlinear mappings between inputs (e.g., chemical descriptors) and outputs (e.g., material properties) [51]. Ideally, a well-trained ML model can uncover underlying physical or chemical principles, and even approximate certain “effective theories” grounded in quantum mechanics [52,53,54].

In recent years, ML has found particularly widespread applications in the field of materials science, especially in the prediction of material properties and the design of novel materials. The construction of effective ML models in this domain depends on two fundamental components: high-quality databases and well-designed descriptors. Databases—such as the Materials Project [55], Automatic-FLOW for Materials Discovery (AFLOW) [56], Open Quantum Materials Database (OQMD) [57], the Open Catalyst Project [58], and PolyInfo [59]—contain extensive material data obtained through experiments or simulations, and serve as the foundation for model training and validation. Descriptors, on the other hand, transform complex information related to atomic, molecular, or polymer structures and compositions into numerical features interpretable by ML models. Ideal descriptors should be unique, discriminative, computable, and physically meaningful. Examples include molecular fingerprints [60], structural similarity indices, topological descriptors, molecular orbital energies, atomic electronegativity, molecular weight, and glass transition temperature [61].

Although traditional ML algorithms—such as support vector machines (SVMs) [62,63], random forests (RFs), and kernel ridge regression (KRR)—have demonstrated success in modeling structured data, they often face limitations when dealing with high-dimensional, unstructured data such as images, speech, or text. This challenge has driven the emergence of deep learning (DL) methods, which offer greater expressive power and generalization capabilities. The turning point came in 2011, when Dan Ciresan’s team achieved a major breakthrough by training DNNs on GPUs for image classification, drawing widespread attention to DL. Building upon earlier efforts by Geoffrey Hinton, Yoshua Bengio, and Yann LeCun, DL employs multilayer nonlinear architectures [64]—such as convolutional neural networks (CNNs), recurrent neural networks (RNNs), and GNNs—to automatically extract and learn hierarchical feature representations from data. These models have proven particularly effective in capturing the complex dependencies within large datasets. The rapid advancement of hardware, the exponential growth of data, and continuous improvements in training algorithms—such as the Adam optimizer, dropout regularization, and batch normalization—have fueled breakthroughs in various domains, including computer vision, natural language processing (NLP), and speech recognition. These successes have, in turn, catalyzed the integration of DL into materials research.

As a result, a diverse array of ML algorithms has emerged (as illustrated in Table 1), offering researchers new tools to accelerate material design [65,66,67,68,69,70,71,72]. In materials science, ML significantly reduces the time and cost of experimental synthesis and characterization, unlocking new possibilities for high-throughput screening and inverse design. DL, in particular, has been widely adopted for property prediction, synthesis route optimization, and generative modeling of new materials. Although training large models demands substantial computational resources, their capacity to handle complex, large-scale datasets gives them a notable advantage. Furthermore, the applications of AI in polymer science continue to expand, encompassing NLP, computer vision, and laboratory automation. Entering the era of Industry 4.0, AI is increasingly being utilized across various stages of materials research, including design, diagnostics, analysis, predictive maintenance, and early fault detection.

### 2.2. The Exploration of Property and Structure Relationship for Polymer Materials

Polymer design and property prediction play significant roles in materials science. With the rapid development of AI, researchers can explore new materials with unprecedented efficiency. In this section, discussions will be conducted on the combination of polymer informatics and the Materials Genome Initiative (MGI), the application of structure–property relationships, and the construction of different design methods and prediction models.

#### 2.2.1. Machine Learning Interatomic Potentials

ML interatomic potentials (MLIPs) have substantially expanded the temporal and spatial scales accessible in polymer simulations. Serving as a critical link between primary polymer structures—including composition, conformation, configuration, and monomer sequence—and macroscopic properties, MLIPs enable efficient, accurate modeling across multiple scales. Conventionally, density functional theory (DFT) offers high precision by solving the Schrödinger equation, but its prohibitive computational cost restricts its use to small systems and short timeframes. In contrast, classical molecular dynamics (MD) simulations, which rely on empirical force fields, are significantly more efficient but often fail to capture complex phenomena involving changes in electronic structure. MLIPs offer a compelling solution to this long-standing trade-off by approaching DFT-level accuracy while maintaining MD-level efficiency [121].

The performance of MLIPs relies heavily on the quality of feature descriptors. Early studies predominantly utilized handcrafted features derived from polymer repeat units or chemical heuristics, with researchers manually identifying correlations between structure and performance from large datasets [122]. However, the rapid expansion of polymer databases has rendered manual feature extraction increasingly unsustainable. To address this, Gurnani et al. introduced advanced DL techniques, including GNNs and multitask learning (MTL), to automatically extract key structural features from polymer repeat units [41]. Compared to traditional handcrafted feature extraction methods, their approach achieved a 1–2 orders of magnitude improvement in feature extraction speed while maintaining comparable prediction accuracy. Further, Zhang et al. proposed the COMFO model, which utilizes three distinct feature extraction methods (the bidirectional encoder transformer model extracts information from simplified molecular input line entry system sequences, the attentive FP network extracts atomic and bond information from molecular graphs, and molecular fingerprints are used to extract substructure information) to accelerate the discovery of polyimide dielectric materials [123]. Compared to traditional feature extraction methods, COMFO achieves a significant improvement in extraction speed while maintaining high prediction accuracy.

**Figure 2:**
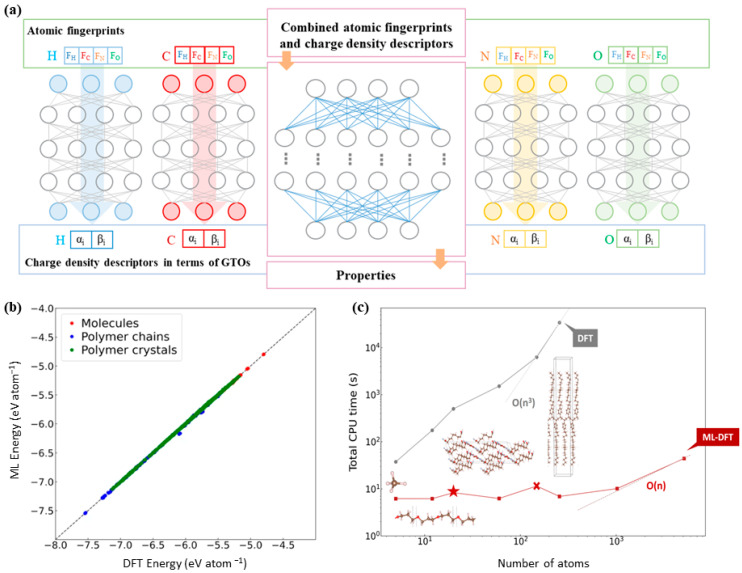
(**a**) Prediction of electron charge density by combining atomic fingerprints with gaussian orbital (GTO) descriptors, and further prediction of other DFT characteristic models using the combined descriptors; (**b**) parity diagram of potential energy for each atom; (**c**) total CPU time of DFT and ML-DFT in electronic structure prediction. This figure is inspired by works from del Rio et al. [124].

**Figure 3:**
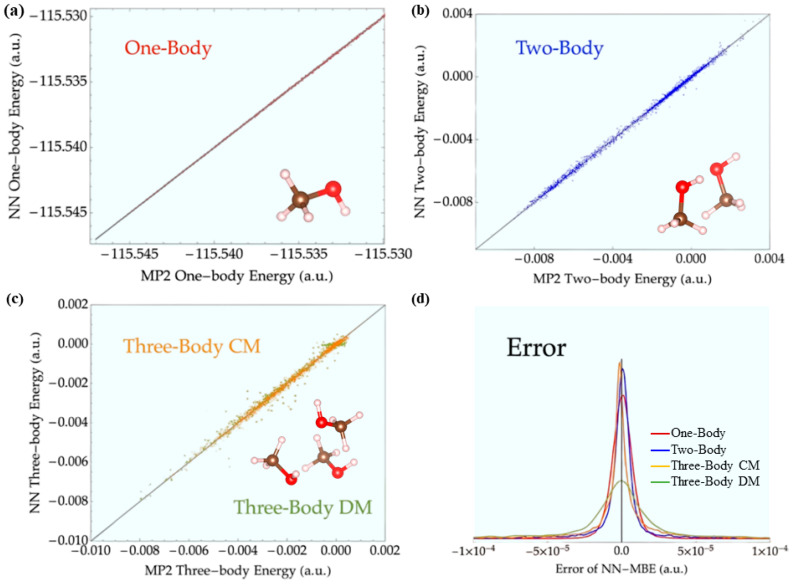
(**a**) From the one-body energy maps calculated by MP2 and neural networks. (**b**) From the two-body energy diagrams calculated by MP2 and neural networks. (**c**) From the three-body energy diagrams calculated by MP2 and neural networks. (**d**) The histogram of the errors of the many-body energy terms predicted by the neural network. This figure is inspired by works from Yao et al. [125].

The other primary factor to determine the performance of MLIPs is the algorithm. To bridge the gap between quantum-level accuracy and large-scale dynamics, some researchers have developed end-to-end learning frameworks that predict electronic charge densities directly from atomic configurations, therefore bypassing the computationally intensive Kohn–Sham equations. A notable example is the work by del Rio et al. (Figure 2), whose model achieves near-ab initio accuracy with dramatically improved computational efficiency, making it particularly suitable for large-scale polymer systems that lie beyond the practical limits of conventional DFT [124]. Another complementary strategy focuses on enhancing MD simulations by incorporating machine-learned or auxiliary energy terms to improve the accuracy of interatomic interactions. For instance, Parkhill and collaborators developed the TensorMol model, which integrates many-body expansions with NNs to account for long-range physical interactions such as Coulombic and dispersion forces, which are often neglected in standard DFT [125]. TensorMol not only reduces computational cost, but also improves the precision and transferability of molecular simulations across diverse polymer systems. Although these approaches provide strong support for bridging quantum-level accuracy and multi-scale modeling, their generalizability across diverse chemical spaces and stability across different material systems remain open challenges. Currently, they are still in the exploratory and optimization stages; nevertheless, they lay a crucial foundation for the future development of universal models that couple electronic structure with mechanical behavior in polymers (Figure 3).

#### 2.2.2. Structure–Property Modeling and Inverse Design of Polymers

Revealing the coupling mechanisms between structures and properties is a key step toward the design of polymer materials with controllable performance and programmable functions. In recent years, structure–property modeling and property-driven inverse design have emerged as transformative paradigms in polymer research.

A group of studies focuses on predicting local responses based on known structures. For instance, Gautham et al. developed a DNN framework incorporating Behler–Parrinello symmetry functions for modeling the potential of mean force between polymer-grafted nanoparticles [126]. This method uses the radial and angular symmetry functions of the nanoparticle’s centroid and grafting points as inputs, and employs a dual-DNN to map the overall interaction potential and local chemical environment potential, effectively capturing the anisotropic interactions induced by the grafted polymers. Although the training data rely on large-scale molecular simulations and have limited generalizability, the method overcomes the limitations of traditional approaches in modeling angle-dependent interactions, significantly improving computational efficiency and laying the foundation for future development of more universal models for interatomic interaction predictions. Shi et al. developed an efficient predictive framework for adhesive free energy from polymer sequences by integrating coarse-grained simulations with support vector regression (SVR) modeling [127]. This framework eliminates the need for complex theoretical assumptions, significantly reducing computational costs. Additionally, by combining GA with the SVR model, the study enables rapid screening of polymer sequences with target properties, providing methodological support for the “inverse engineering” of functional polymers. Although the framework has limitations in generalization capability and adaptation to real-world systems, it is expected to be further extended to diverse interfacial material designs. Yang et al. developed a Fiber Network Deep Learning System that extracts the structural and mechanical information of fiber networks from atomic force microscope images using DL [128]. This method addresses the challenge of low-quality image segmentation, enabling the identification of parameters such as fiber contour length and node distribution. By combining the worm-chain model to calculate mechanical indicators like persistence length, the method was validated in silk fibroin nanofibers. This work lays the foundation for achieving the ultimate goal of “image-driven material design.” By extracting chemical-independent features of block polymers from small-angle X-ray scattering (SAXS) data and applying a physics-informed ML algorithm, Fang et al. achieved out-of-sample predictive accuracy of around 95% over the identification of morphologies [129]. Collectively, these efforts provide efficient and deployable structure–property models that make the prediction of complex mechanical and interfacial behaviors possible, significantly improving performance evaluation and experimental planning.

Another line of research takes a further step forward by reversing the modeling direction: from target properties back to optimal material structures—inverse design [130]. Given the vast design space of polymers, such as monomer sequences and component combinations, exhaustive search is intractable. Data-driven inverse design thus offers a new route for performance-oriented structural optimization. For example, Li et al. achieved intelligent regulation of polymer molecular weight distributions through the deep integration of RL and atom transfer radical polymerization simulations, providing efficient and scalable strategies for the synthesis of functional materials [131]. Despite challenges related to model simplifications and limited experimental validation, their demonstrated AI-driven synthesis approach paves a new path for cross-disciplinary research in polymer science and ML, offering a new paradigm for “inverse synthetic design.” Kranthiraja et al. demonstrated how to use a random forest model to develop organic photovoltaics with a relatively small number of experimental data points (566) and screen a large number of molecular structures [132]. Although issues regarding descriptors and model interpretability still persist, they presented an experimental-oriented, data-driven research paradigm, offering new ideas for research in this field. Webb et al. achieved targeted polymer sequence design through the deep integration of coarse-grained modeling and ML [133]. This paradigm not only demonstrates prediction accuracy exceeding 95% for target properties and universality in cross-class polymer design, but also enables a substantial reduction in computational cost and ensures target deviation within 10%, proving the significant advantages of data-driven approaches in complex molecular systems. This framework provides critical technological support for overcoming bottlenecks in polymer design and is poised to accelerate the translation of theoretical designs into practical applications for high-performance materials. Hiraide et al. constructed the first DL-based inverse design framework for phase-separated structures in polymer alloys through the collaboration of conditional generative adversarial networks (cGANs) and CNNs, enabling the inference of optimal microstructures from target mechanical stresses [134]. The framework demonstrates excellent performance in both structural generation accuracy (structural factor matching) and mechanical property prediction accuracy (R^2^ > 0.9), providing an efficient and scalable methodology for the rational design of high-performance materials. This approach holds promise to drive a paradigm shift in materials science from “trial-and-error experiments” to “data-driven design.”

**Figure 4:**
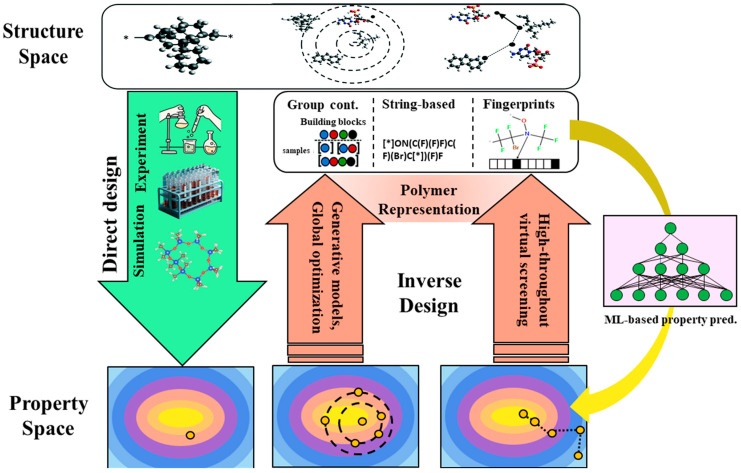
Schematic diagrams of forward and reverse material design. This figure is inspired by works from Sattari et al. [136].

Meanwhile, Zhu et al. constructed a “data-driven soft sensor” using a deep belief network (DBN) model, demonstrating excellent performance in nonlinear and data-imbalanced industrial scenarios [135]. This provides an efficient and reliable solution for real-time quality control in polymer production processes, marking a significant application breakthrough of DL in chemical process modeling. Sattari et al. proposed a data-integrated inverse design framework that combines simulation and experimental data, enabling efficient closed-loop optimization from design to manufacturing, offering promising solutions for rapid development of functional polymers (Figure 4) [136].

#### 2.2.3. Properties Prediction Model

In the modeling framework of structure–property relationships for polymeric materials, beyond the structure-induced local response behavior, an alternative yet equally critical research direction is the ML-based prediction of intrinsic properties. Intrinsic properties refer to macroscopic physical or chemical characteristics exhibited by polymers under standard conditions, such as glass transition temperature (Tg), electronic bandgap, Young’s modulus, dielectric constant, melt flow index, and gas permeability. These properties often determine the applicability, safety, and functional performance of materials in specific engineering contexts. The central objective of intrinsic property prediction is to construct accurate and generalizable mappings from molecular inputs—such as SMILES strings, molecular fingerprints, or graph-based representations—to scalar property outputs [137]. Such models aim to replace time-consuming and costly experimental or computational procedures, thereby enabling high-throughput screening and performance-oriented material design.

A representative early work in this area was reported by Pilania et al., who utilized the RF and KRR models to study polyhydroxyalkanoate (PHA) polymers and established the mapping relationship between their structures and the Tg [138]. This model demonstrated high prediction accuracy. Although this research method has certain limitations in considering the influence of polymer configuration and expanding the application scope, it is superior to traditional methods in terms of prediction accuracy and other aspects, and it is also universal. To further enhance the adaptability and transferability of the model, Shi et al. utilized transfer learning techniques and significantly improved the accuracy of the GNN model pre-trained on shorter oligomers (with errors of excited-state energy that can be less than 200 meV compared to time-dependent density functional theory calculations), even with only a small dataset of longer oligomers, thus solving the problem of insufficient training data in the modeling of optoelectronic properties of long conjugated oligomers and polymers [139].

MTL has also emerged as an effective strategy to improve modeling efficiency and capture inter-property correlations. Kuenneth et al. used a multitask DNN to predict 13 polymer properties, covering thermal, mechanical, and gas permeability aspects [140]. The model leverages data sharing to improve training efficiency and shows good prediction performance. However, there are potential problems in reconciling multi-objective weights. If not properly handled, it will reduce the performance of individual tasks, and future research is needed to explore optimization strategies. This challenge may be mitigated by multi-origin data integration strategies enabled by transfer learning. As demonstrated in pioneering work by Phan et al. [141], language models pre-trained on quantum chemical datasets of small molecules can be effectively fine-tuned using sparse experimental measurements of polymeric macroscopic properties. This approach leverages cross-scale knowledge transfer: the model first captures fundamental physics from computationally tractable systems, then extrapolates to complex polymer behaviors by domain adaptation. Crucially, such frameworks overcome data scarcity by establishing hierarchical feature relationships between theoretically accessible properties (e.g., dipole moments influencing permittivity) and experimentally measured bulk characteristics (e.g., mechanical strength). This paradigm represents a fundamental advancement beyond conventional simulation-reliant approaches. Similarly, Himanshu et al. proposed a method for selecting NN topologies through layer-by-layer expansion and a data selection method based on the latent space, which effectively solved the key problems of DL models in predicting polymer properties [142]. These two methods can be extended to other polymer and material systems and can be further combined to form an efficient workflow, which is of great significance for the feature learning of polymers and other materials and the development of ML models.

**Figure 5:**
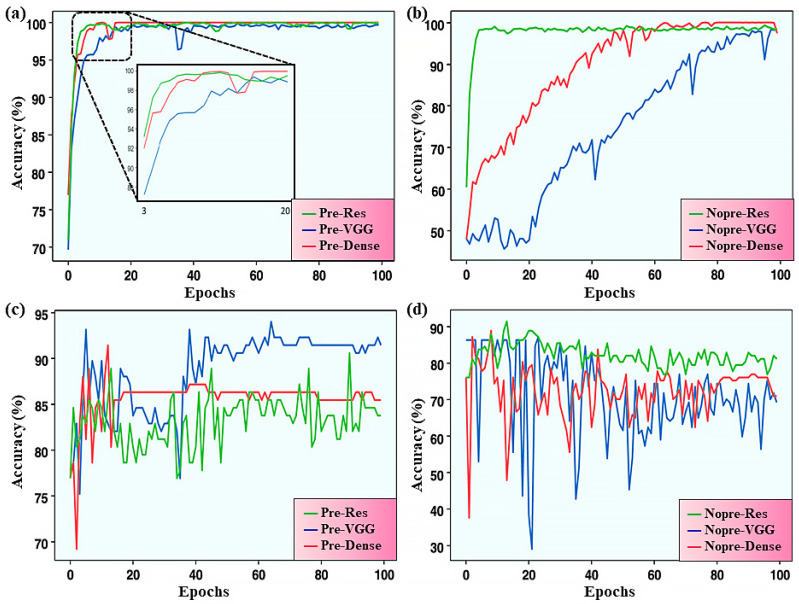
Accuracy of the models on the training set and test set in the training process. (**a**) Accuracy of pretrained models on the training set. (**b**) Accuracy of non-pretrained models on the training set. (**c**) Accuracy of pretrained models on the test set. (**d**) Accuracy of non-pretrained models on the test set. This figure is inspired by works from Liang et al. [143].

Beyond structure-based representations, some studies have explored image-based features for property inference. For instance, Liang et al. utilized convolutional NNs and transfer learning methods to achieve automatic miscibility identification [143]. The accuracy of the model reached as high as 94%, and they also proposed a quantitative criterion for polymer miscibility using this model. Although the performance of this method is limited by the quality and clarity of SEM images, with the increase in the dataset, it is expected that this problem can be solved in the future (Figure 5). Moreover, the success of the MGI has brought new opportunities. Taking advantage of this, Kim et al. developed a fingerprinting method that can capture polymer characteristics from the atomic to the morphological and structural levels [144]. This method, combined with ML models, can rapidly predict polymer properties. The related models have been integrated into the Polymer Genome online platform. At the same time, the research team also plans to expand the chemical and property space to further enhance the platform’s support for polymer material research in different fields. By applying evidential neural networks on light scattering and SAXS data of single-chain polymer nanoparticles, Upadhya et al. successfully predicted, synthesized, and characterized 30 novel compact SCNPs, where 58% of them had a Porod exponent ≥ 3.5 [145].

In summary, intrinsic property prediction constitutes a “precision modeling” pathway for the application of ML in polymer science [146]. Emphasizing prediction accuracy, structural representation fidelity, and inter-system generalization, these approaches provide a robust foundation for material discovery and targeted optimization.

### 2.3. Polymer Process Optimization

In the research and development of polymer materials, the high cost of experimental resources, long synthesis cycles, and vast parameter spaces are the core bottlenecks restricting the rapid discovery of high-performance materials. In recent years, AI technology has gradually entered the experimental stage [147]. It is no longer merely used for predicting material properties or evaluating the quality of structures, but is directly integrated into the entire process of experimental design, parameter optimization, and even synthesis decision-making. This has promoted a transformation in the experimental paradigm from “model-assisted” to “system-led”. Depending on the optimization objectives and the depth of intervention, existing research can be roughly divided into two categories: parameter optimization with an unchanged process and system-level experimental closed-loop iteration.

#### 2.3.1. Parameter Optimization of the Process

Prior to the widespread adoption of AI, statistical process control and design of experiments (DOE) were the dominant strategies for process optimization in polymer materials. However, these traditional approaches often suffer from long experimental cycles, complex data processing, high costs, limited generalizability, and a lack of ability to capture nonlinear interactions among multiple variables. With the advancement of AI technologies, their integration with polymer science has introduced transformative solutions [147]. Leveraging ML algorithms such as SVMs and neural NNs, researchers have developed data-driven models that enable intelligent optimization of input parameters without altering the underlying experimental or synthetic protocols. These models have significantly improved experimental efficiency and product performance.

ML-based parameter optimization has now become a critical component of intelligent polymer design. Depending on their specific goals, such efforts can be broadly classified into two categories: (1) process-level parameter control, with a strong focus on polymer forming such as additive manufacturing techniques, and (2) structural-level parameter regulation targeting material composition and macroscopic performance.

In the field of additive manufacturing, Khanzadeh et al. employed the self-organizing map method to analyze geometric error patterns in the fused filament fabrication (FFF) process of polymers [148]. By processing point cloud data, this method effectively identified various types of deviations without requiring manual annotation. The study demonstrated that geometric accuracy could be accurately characterized using only 2.4% of the total data points, significantly accelerating scanning and defect detection. Although the research was based on simplified test parts, which limits its generalizability to complex geometries, it laid a solid foundation for the analysis of more intricate shapes and fine features.

In the area of thermal field modeling, the NN model developed by Roy et al. demonstrated exceptionally high computational efficiency [149]. Compared with traditional finite element simulations, the model required only 0.036 s to generate a single temperature curve, with a prediction error of less than 5%. It exhibited strong stability and applicability in FFF processes with fixed structures and processing conditions, providing valuable support for optimization, process planning, and real-time monitoring in closed-loop control during 3D printing.

**Figure 6:**
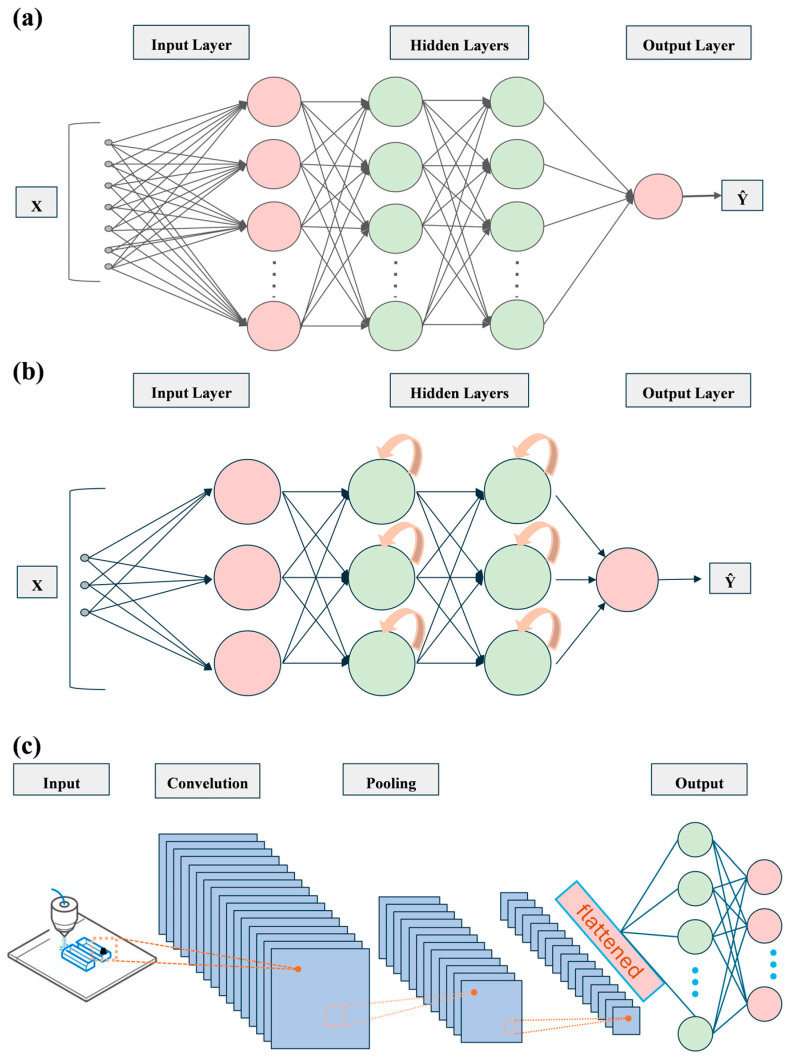
General architectures of different DL models. This figure is inspired by works from Nasrin et al. [155]. (**a**) Multilayer perceptron for predicting polymer AM print properties; (**b**) RNN for quality control in polymer AM prints; (**c**) CNN for defect detection in a polymer AM process.

Beyond these examples, ML has also played a significant role in controlling other processes such as material extrusion, photopolymerization, powder bed fusion, binder jetting, and material jetting. For instance, Zhu et al. used principal component analysis (PCA) and SVM to reduce printing defects [150]; Edlim et al. applied a CNN based on the you only look once (YOLO) architecture for anomaly detection [151]; and Lee et al. utilized 3D-CNN and CNN-LSTM models to identify defects in two-photon lithography [152]. In addition, Sassman et al. used SVM to develop a pre-screening method based on powder flowability and surface roughness [153], while Satterlee et al. combined image enhancement with various CNN architectures to detect porosity-related defects [154]. These studies collectively demonstrate the strong adaptability and broad applicability of DL models—including multilayer perceptrons (MLP), RNNs, and CNNs—in polymer forming. Representative architectures and their primary applications are illustrated in Figure 6.

Beyond polymer forming, parameter optimization has also been widely applied to the regulation of material composition and performance. Chen employed an integrated ML strategy combined with the Sure Independence Screening and Sparsifying Operator (SISSO) method to analyze experimental data, optimize the dispersion ratio of graphene oxide–hydrogen-bonded zinc hydroxystannate in a polypropylene (PP) matrix, and developed a predictive model for flame retardancy [156]. This approach significantly improved the flame-retardant properties of the PP composite and shows strong generalizability, with the potential to be extended to other polymer systems.

Li et al. applied Bayesian optimization (BO) to tune the synthesis conditions of short polymer fibers [157]. Their study demonstrated that BO is capable of efficiently searching sparse process spaces and substantially reducing the number of experiments, outperforming traditional grid search methods. However, its applicability to other reaction systems remains to be further explored. Similarly, Takasuka et al. used a flow synthesis system to prepare styrene–methyl methacrylate copolymers and employed BO to adjust processing variables, confirming the method’s effectiveness in regulating monomer ratios in copolymers [158]. In the future, multi-objective BO is expected to enable the simultaneous optimization of copolymer composition and physical properties, further advancing the intelligent design of copolymers.

In summary, parameter optimization strategies have undergone a paradigm shift in polymer research, from early-stage unsupervised clustering and statistical regression to advanced techniques such as BO, MTL, and hierarchical modeling. These methods have demonstrated exceptional performance in structured, parameter-sensitive processes like additive manufacturing, and also provide robust technical support for broader applications in copolymer design, composite formulation, and coarse-grained parameter calibration [159,160].

#### 2.3.2. Process Iteration and Experimental Closed-Loop Systems

Building on previous advances in parameter tuning and process control, recent research has turned to the development of automated, closed-loop systems that enable iterative experimentation, intelligent decision-making, and real-time optimization in polymer materials engineering. In recent years, the continuous advancement of AI and automation technologies has profoundly impacted polymer materials engineering, particularly in enhancing experimental efficiency, precision control, and sustainability [161,162]. The integration of DL algorithms with high-throughput experimental platforms has played a critical role in the construction of process iteration and experimental closed-loop systems, gradually reshaping traditional materials research paradigms.

In the domain of material identification and sorting, Sbrana et al. combined near-infrared hyperspectral imaging technology with the N-BEATS DL model to classify over 4500 plastic samples [163]. The overall classification accuracy of the model reached 79%, with 90% accuracy for colored plastics and 67% for black plastics. This data-driven approach has significantly improved recycling efficiency in material application scenarios, promoting the optimization of raw material procurement and the upstream links of the supply chain.

In polymerization reaction research, Rizkin et al. used a semi-autonomous microfluidic platform combined with in situ thermography and NN to gain a deeper understanding of the catalytic cycle of homogeneous polymerization reactions [164]. The study demonstrated the feasibility of integrating high-throughput microfluidic technology with ML algorithms, reducing chemical waste and energy input. While the reaction system had certain limitations, it significantly improved experimental efficiency and greatly reduced chemical waste in the zirconocene-catalyzed α-olefin polymerization reaction system, opening up new avenues for polymerization reaction research.

Furthermore, process automation has emerged as a key enabler of high-throughput experimentation. Cao et al. developed an ML-guided experimental platform that integrates BO with DOE strategies [165]. The platform, applied to the optimization of complex liquid formulations, conducted hundreds of experiments within 15 working days and identified nine optimal candidate formulations that met all predefined specifications. Compared to traditional trial-and-error workflows, this approach significantly improved both time efficiency and prediction accuracy.

**Figure 7:**
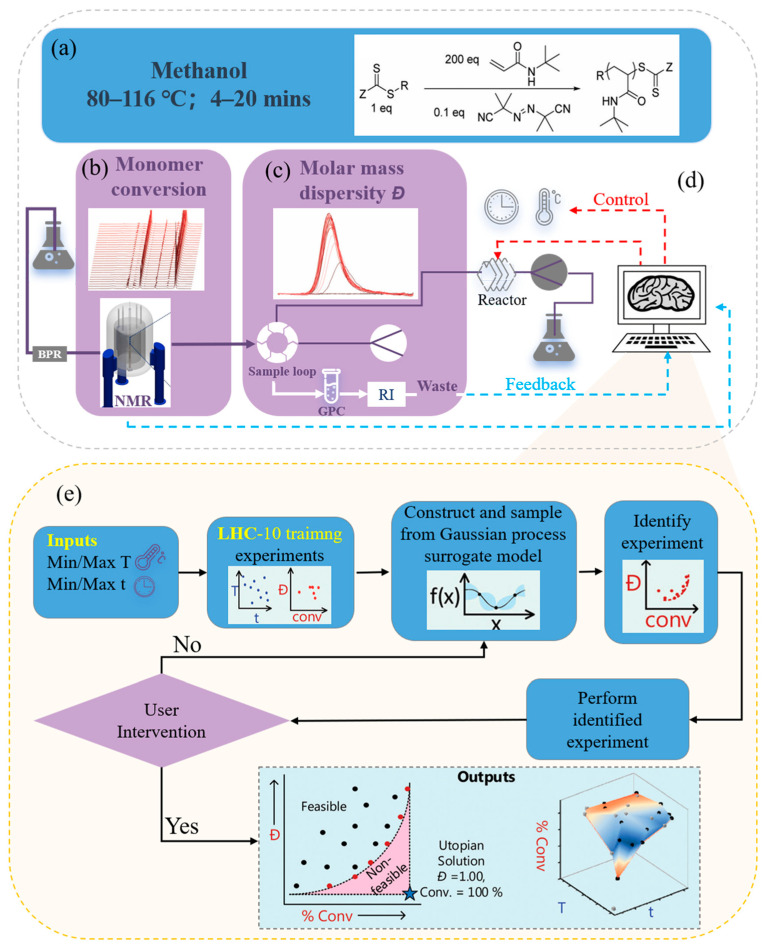
Schematic diagram of a multifunctional fully automatic synthesis platform taking the programmed RAFT polymerization of tert-butylpropanamide as an example. This figure is inspired by works from Knox et al. [166]. (**a**) Generalised scheme for the RAFT synthesis of P(tBuAm)_200_. Example; (**b**) Hydrogen nuclear magnetic resonance (NMR) spectra from the automated continuous flow platform (see ESI for full platform details and all analytical data); (**c**) Gel permeation chromatography hydrogen nuclear magnetic resonance (NMR) spectra from the automated continuous flow platform (see ESI for full platform details and all analytical data); (**d**) Schematic of the automated platform; (**e**) Overview of the machine learning-directed experimental setup using the Thompson-sampling efficient multi-objective optimisation (TSEMO) algorithm.

To further enhance synthesis precision and adaptability, Knox et al. successfully developed a platform capable of conducting automated reversible addition-fragmentation chain transfer polymerization under special conditions (Figure 7) [166]. The TSEMO algorithm effectively reduced the number of experiments and achieved closed-loop multi-objective optimization. This technology has broad application prospects in various polymerization techniques with more input variables and objectives, and follow-up research can further clarify the properties of polymers produced under extreme conditions.

In summary, process iteration and experimental closed-loop platforms are emerging as foundational tools in the transition of polymer research from empirical paradigms to data-driven strategies. By tightly integrating experimental design, real-time feedback, algorithmic decision-making, and robotic execution, these platforms address long-standing inefficiencies in materials development [167,168].

## 3. Challenges and Feasible Solutions

AI has demonstrated promising potential in polymer research including molecular structure design, property prediction, process parameter optimization, and the development of intelligent closed-loop laboratories. These advances are gradually driving the transformation of materials research from an experience-driven to a data-driven paradigm. However, compared to other material systems, the application of AI in polymers remains in its early exploratory stages [169]. The current challenges include: (1) the scarcity of large-scale, high-quality, structured, and representative datasets; (2) the lack of universal descriptor systems capable of effectively representing the multi-scale structures of polymers; and (3) the need for optimization of model architectures and training mechanisms.

### 3.1. Databases

The construction and utilization of data have become the main bottleneck restricting the further development of this field. Compared with relatively simple systems such as inorganic materials and two-dimensional materials, polymer materials face greater challenges in data acquisition due to their complex molecular structures, diverse synthesis routes, and long experimental cycles. Although the early datasets established by researchers such as Treloar [170] provided valuable references, their limited sample sizes and low dimensions can no longer meet the growing demands of modern large-scale models for data diversity and quantity.

The current challenges mainly focus on two aspects: (1) the high cost of data acquisition makes it difficult to collect large-scale data; and (2) there are significant inconsistencies in formats and measurement standards, increasing the difficulty of data cleaning and integration. In this regard, the successful experiences in fields such as semiconductors, metals, and biomaterials are particularly important [171]. These fields have made remarkable progress in reducing data acquisition costs and unifying formats and measurement standards, providing important references for the polymer materials field. To address the above issues, we can learn from three aspects: data generation, potential data mining, and database construction.

In terms of data generation, the combination of high-throughput experiments and ML has shown great potential. For example, the research team led by Zhang proposed an entropy-based active learning (ET-AL) framework to alleviate the structure–stability bias in material data, especially achieving good results on datasets obtained from DFT calculations [172]. This method uses information entropy as a diversity metric. For the OQMD-8 dataset (containing binary alloy data of elements such as aluminum and titanium) and the J-CFID dataset (containing elastic modulus data), it enhances the diversity of underrepresented regions through AL, significantly improving the accuracy of ML models in predicting material properties (such as bulk modulus and shear modulus) while reducing data bias. This approach provides a new path for data-driven modeling in material discovery. Similar strategies can be applied to the polymer field, combined with high-throughput experiments and ML algorithms, to improve the prediction ability of polymer material properties.

At the same time, multitask learning based on simulated data has become an important supplement to experimental data. For example, the method of Hamiltonian parameterization for perovskite materials proposed by Ma et al. enables a seamless transition from DFT data to large-scale atomic simulations through AL [173]. This research effectively deals with the complex interactions in the complex perovskite system. Polymer systems also have complex interactions such as inter-chain interactions and side-group interactions. The success of this model provides a new way to handle the complex interactions in polymer systems and simulate their structures and behaviors in different environments. In addition, adversarial algorithms have also shown significant potential in enhancing model robustness and enriching data diversity. For example, Yang et al. proposed a two-step data augmentation method based on the GAN model for predicting the hardness of high-entropy alloys, and revealed the feature importance and the reasons for the performance improvement of the two-step method through interpretability analysis [174]. By referring to the two-step data augmentation method, generating polymer data similar to the real data distribution through the generative adversarial network can expand the dataset. When studying the properties of new polymer materials, this method can be used to generate more data, reduce the impact of insufficient data on the model, and improve the prediction accuracy of the model.

In terms of potential data mining, a large number of experimental parameters and performance records in academic literature can be used as important supplementary resources. A large number of experimental parameters and performance records are scattered in scientific publications. Through NLP tools and large language models (LLMs), this information can be efficiently extracted and structured. For example, the ChemMatch framework proposed by Xiuying Chen et al. demonstrates the potential of literature-driven dataset construction, significantly expanding the database capacity while reducing the cost and cycle of experimental data acquisition [175]. This method provides a reference for related research in the polymer field. However, special attention should be paid to the accuracy and semantic integrity when extracting unstructured data such as images and charts to prevent the loss of valuable information during the conversion process. Yan et al. proposed a semi-supervised material information extraction framework based on an automatically generated corpus [176]. They used Snorkel to automatically annotate the corpus and trained the model with an ON-LSTM network, showing good performance in multiple material property extraction tasks, providing a new method for information extraction in the material field. Although there are currently problems such as a small number of datasets and imbalanced positive and negative samples, the idea of automatically annotating corpora has certain reference significance for processing material literature containing images and charts.

In addition, the development of databases has brought new opportunities to solve data-related problems in the polymer field. Although research institutions in various countries have actively established databases related to polymers (as shown in Table 2), there is still a lack of a widely recognized large-scale authoritative platform like The Materials Project in this field. While specialized databases remain essential for application-specific challenges, universal polymer databases would provide complementary foundational value. Such platforms would serve two critical functions: (1) revealing intrinsic structure–property relationships through systematic cross-material comparisons, and (2) enabling transfer learning approaches where large language models pre-trained on universal datasets are fine-tuned using domain-specific experimental data. This hierarchical ecosystem, with universal databases scaffolding specialized repositories, creates pathways for AI acceleration comparable to advances in inorganic materials science. When building such a platform, it is necessary to develop clear data collection standards, metadata specifications, privacy protection measures, and access control systems to improve professionalism and sustainability and enhance data utility. Therefore, promoting interdisciplinary cooperation and establishing a standardized polymer database system is an important direction for future development. In addition, building online resource libraries [177,178] to improve data accessibility and standardization is also an important means. However, attention should be paid to solving problems such as data maintenance, updates, and access rights management. A perfect operation and maintenance mechanism should be developed, and interdisciplinary alliances should be promoted to establish unified data standards and privacy protection guidelines to ensure the long-term operation of the database.

In conclusion, to achieve a major breakthrough in the application of AI in polymer materials, it is necessary to address fundamental issues such as limited data availability, insufficient data quality, and inconsistent standards. Only by building an open, standardized, and high-quality data infrastructure can we provide solid support for AI model training and fully unleash the potential of AI in polymer design, property prediction, and process optimization.

### 3.2. Descriptors

Polymer materials typically exhibit characteristics such as a wide distribution of molecular weights, complex three-dimensional conformations, branching and cross-linking structures, and inter-chain entanglements. These inherent structural complexities make it extremely difficult to accurately capture their microscopic features and effectively map them to macroscopic properties.

The challenges regarding descriptors mainly focus on the following three aspects: (1) given the diversity of polymer structures, it is difficult to construct effective descriptors; (2) high-dimensional descriptors are prone to the “curse of dimensionality”; and (3) there are significant differences in the sources and scales of descriptors, leading to difficulties in integration.

The root cause of these challenges lies in the inherent structural complexity of polymer materials. Compared to crystalline metals or inorganic semiconductors with relatively ordered structures, polymer chains exhibit a wide range of features such as backbone compositions, branching structures, copolymer sequences, and hierarchical features like cross-linking points, crystalline regions, and amorphous regions. This complexity makes traditional low-dimensional or statistically simple features insufficient for performance modeling. For example, in branched polymers, subtle differences in the number, length, or position of side chains can significantly affect mechanical, thermal, or solubility properties. Encoding these subtle yet important features into machine-readable structured descriptors remains a huge challenge. To address these issues, we will elaborate from three aspects, descriptor construction, dimensionality reduction and feature compression, and standardized descriptor systems, drawing on experiences from other fields.

In terms of descriptor construction, research in the field of protein science provides important references for the polymer field. For example, in the case of sequence order, Yu and his colleagues did not directly use the composition of peptide chains as features [179]. Instead, they assigned each peptide chain to a structural category based on its structural class trends and then transformed the protein sequence into a sequence of structural category trends, which improved the prediction accuracy. Similarly, for polymer materials, the monomer sequence in the polymer chain can be used as a similar feature. This helps to reveal the deep-seated relationship between the polymer structure and its properties. Although polymers are more complex than proteins in terms of spatial freedom and inter-chain interactions, this analogy provides valuable inspiration for the design of polymer-specific descriptor systems.

To improve the expressiveness and adaptability of descriptors, innovation has been attempted from two aspects: multi-scale modeling and time-series analysis. Considering that materials often undergo dynamic evolution during use—such as thermal aging, mechanical fatigue, or environmental responses—the design of descriptors with a time dimension has become a focus of attention. In recent years, sequence models such as long short-term memory (LSTM) and Transformer have been used to capture the time-dependent evolution of materials. These methods enhance the time-resolution ability of descriptors and provide new ideas for predicting non-equilibrium behaviors, service life, and durability. For example, Niu evaluated the performance of four different neural network architectures—MLP, CNN, LSTM, and Transformer—in predicting the Vickers hardness of M50NiL steel [180]. The study found that MLP performed outstandingly in prediction accuracy and iterative efficiency. Meanwhile, LSTM also showed high accuracy in predicting Vickers hardness and had certain advantages in handling data with time-series characteristics or sequential patterns, which may be helpful for analyzing the related properties of complex materials. Inspired by this, Lee’s team applied LSTM and feed-forward neural network (FNN) deep-learning models to predict the tensile behavior of polymer–matrix composites (PMC), especially achieving progress in predicting the tensile failure point [181]. However, despite these important advancements in the field, there are still key issues such as data uncertainty, how to handle high-dimensional data, and eliminating redundant features.

To address the problem of dimensionality caused by high-dimensional features, researchers have explored dimensionality reduction and feature compression techniques. Bahoria et al. proposed a model based on a hybrid DL and GA approach for optimizing and predicting the compressive strength of concrete [182]. In this process, PCA was applied to reduce the high-dimensional feature set to 150 principal components, reducing the feature dimension by approximately 65% while retaining 95% of the data variance. This dimensionality reduction method not only significantly reduced the computational burden, but also effectively alleviated the overfitting problem and improved the generalization ability of the model on the training and validation sets. This strategy is helpful for eliminating redundant variables, reducing training costs, and enhancing the interpretability and robustness of the model, which also has reference significance for the polymer field.

Finally, the standardization of descriptor systems remains an important but unresolved issue. Currently, many research teams rely on custom-made or field-specific descriptor frameworks, which leads to difficulties in result comparison, data sharing, and model transfer. To solve this problem, polymer informatics can draw on the standardization efforts in the broader field of materials informatics, such as ISO standards, AFLOWLIB metadata schemas, and MatML protocols, and promote the development of a unified descriptor-encoding system based on physical and chemical properties. Through initiatives by international materials database consortia and professional working groups, a standardized descriptor library and data-sharing platform for various polymer systems can be established. This will significantly enhance the accumulation and transfer of research results and greatly promote the universality, reproducibility, and collaborative potential of AI models in polymer science.

In summary, the key to enhancing the predictive modeling ability of polymer materials lies in a deep understanding of their structural complexity and dynamic characteristics. Constructing hierarchical, multi-scale, and time-resolved structured descriptors—combined with dimensionality reduction and nonlinear modeling techniques—can significantly improve modeling efficiency and expressiveness. At the same time, promoting descriptor standardization will lay a solid foundation for the wide application of AI in polymer design, thus supporting the development of new materials and accelerating technological deployment.

### 3.3. Task-Orientated Algorithm

At the model level, there are still two core challenges that hinder the further development and practical application of AI in polymer science: (1) the lack of model interpretability; and (2) high computational costs associated with scalability.

**Figure 8:**
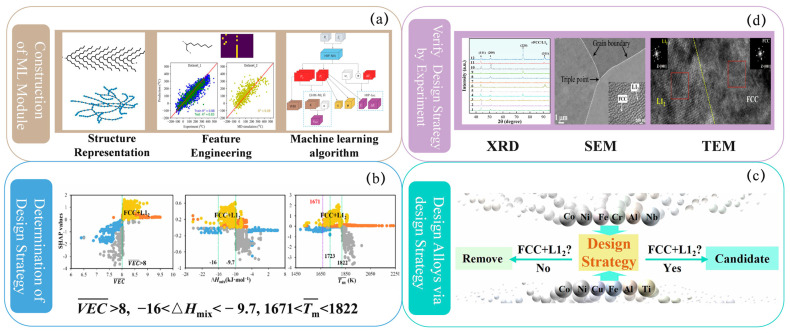
Divided into four parts: (**a**) construction of machine learning models; (**b**) determination of design strategy; (**c**) design alloys via design strategy; (**d**) verify design strategy by experiments. This figure is inspired by works from Tao et al. [183].

Firstly, the “black-box” nature of AI models remains a major obstacle to their credibility and application in polymer material research. Unlike traditional theoretical models based on clear physical principles, although DL models have powerful nonlinear fitting capabilities, they often fail to provide transparent decision-making logic or causal explanations, especially in complex multi-scale polymer systems. To address this issue, the materials science community has widely adopted general interpretable AI methods such as Shapley Additive Explanation (SHAP) and Local Interpretable Model-Agnostic Explanation (LIME). SHAP can conduct global feature importance analysis to help identify the key structural factors determining material properties, while LIME focuses on local interpretability, which is helpful for experimental design and anomaly detection. Tao and his team proposed a model-based knowledge extraction method for the design of multi-principal-element high-temperature alloys (MPESAs) [183]. They constructed two classification models to predict phase composition and used the SHAP method to extract knowledge and transform it into material rules (Figure 8). After verification, the prediction accuracy of these rules exceeded 98%, enabling the rapid design of alloys with an “FCC + L1_2_” dual-phase structure. Twelve alloys prepared experimentally all exhibited the ideal dual-phase microstructure, verifying the accuracy of the strategy. In the polymer field, similar methods can be borrowed to predict the microstructure and properties of polymers. For example, a multi-scale model of polymers can be constructed, and methods such as SHAP can be used to extract the key structural factors affecting performance. This can not only improve the efficiency of polymer design, but also provide a scientific basis for optimizing the functionality and processability of polymers.

In addition, symbolic regression (SR) has recently attracted attention as a modeling technique, capable of learning explicit mathematical expressions that map structures to properties. For example, Yang et al. used the SR method to find a new mixing-length formula that is generally valid in wall-bounded turbulence [184]. This formula has a physical interpretation and has the correct asymptotic relationships in the viscous sub-layer, buffer layer, logarithmic-law region, and outer region. Its accuracy and generalization were demonstrated through classical cases, showing excellent interpretability and physical expressiveness. Although the application of SR in polymer modeling is still in its infancy, its advantages—such as a transparent expression format and controllable physical consistency—make it have great potential in revealing hidden structure–property mechanisms, especially for generating scientific hypotheses and modeling fundamental principles. Such methods will be crucial for improving the physical credibility and scientific acceptance of AI models in this field.

Secondly, the high computational requirements of AI models are another key obstacle to their widespread application in polymer research. The inherent structural complexity of polymer chains and their wide conformational space pose a huge challenge to computational resources when training deep models with large-scale high-dimensional data. In many industrial applications, the limitations of GPU capacity and memory availability pose serious challenges to model deployment. To address this issue, researchers are increasingly focusing on lightweight model design strategies that improve computational efficiency at the architectural level. Linear sparse modeling techniques, such as Least Absolute Shrinkage and Selection Operator (LASSO) and SISSO, have shown excellent performance in feature selection and dimensionality reduction. For example, the Ouyang team applied the SISSO method to classify the metal/insulator characteristics in multiple binary systems and achieved an accuracy of 99.0% [185]. This method also showed high accuracy in predicting the enthalpy difference between rock-salt and zinc-blende structures. However, misclassifications were still observed for some compounds. This method significantly reduced the size of the feature space, thereby reducing the computational burden. These techniques effectively reduce model complexity and training costs while maintaining prediction accuracy. Although they may have certain limitations in capturing the highly nonlinear structure–property relationships in polymer systems, they remain valuable tools in feature engineering and model pre-processing.

In summary, AI models in polymer material research still face key challenges such as computational burden and lack of interpretability. Future work should focus on developing lightweight and efficient modeling strategies while establishing an interpretable AI framework for the context of polymer science. These advancements will play a crucial role in achieving more transparent, efficient, and reliable AI-assisted workflows in material discovery and development.

## 4. Summary and Prospect

This paper outlines the achievements and challenges of AI, polymer research, and proposes feasible solutions. The application of AI in the polymer materials domain has made significant progress, especially in areas such as material design, property prediction, and process optimization, demonstrating immense potential and strongly driving the field’s shift from an experience-driven to a data-driven model.

However, despite the strong application potential of AI in the polymer materials field, numerous challenges remain. Specifically, in terms of data, there are high costs for data acquisition in the polymer field, a lack of high-quality datasets, and the absence of mature and comprehensive data platforms. In terms of descriptors, the complex structure of polymers makes the construction of effective descriptors difficult, and there are also issues with dimensionality and inconsistent standards. In terms of models, due to the uniqueness of polymer structures, existing ML models generally suffer from poor interpretability and high computational costs. To address these challenges, strategies like AL, LLM data mining, time-serious model adoption, PCA dimension reduction, descriptor standardization, SHAP and LIME techniques, and SR methods, drawn from experiences in other fields, can be adopted.

Looking forward, the integration of AI with polymer materials will develop in a more efficient, intelligent, and sustainable direction. As AI technology continues to evolve and improve, it will play an increasingly important role in the design, prediction, and optimization of polymer materials. Solving the challenges related to data, descriptors, and models will be the key direction of future research. Moreover, promoting interdisciplinary collaboration will accelerate this process, bringing more innovative solutions to the polymer materials field. In this process, AI will not only be a tool for polymer research, but also the core driving force behind revolutionary changes in the polymer field.

## Figures and Tables

**Table 1:** Classification of machine learning algorithms and their classic and latest developments.

	Class of Algorithm	Classic Important Algorithms	Latest Development Algorithm
Machine learning	Supervised learning	Linear model [73], logistic regression [68], decision tree [74,75], support vector machine [76], random forest [65], gaussian process regression [77,78], multilayer perceptron [79]	XGBoost [80], LightGBM [81], CatBoost [82], TabNet [83], Neural Tangent Kernel [84]
	Unsupervised learning	K-means [85], hierarchical clustering [86], principal component analysis [87]	UMAP [88], Deep clustering [89], Variational autoencoder [90,91], learning contrast (SimCLR [92], MoCo [93])
	Semi-supervised learning	Self-training [94], co-training [95], label propagation [96]	FixMatch [97], MixMatch [98], Pseudo-labeling [99], Snorkel [100], Consistency Regularization [101]
	Deep learning	Convolutional neural network [102], recurrent neural network [103], long short-term memory [104], generative adversarial network [105]	Transformer [106] (BERT, GPT, Vision Transformer), Diffusion model [107], Graph Neural Networks [108]
	Reinforcement learning	Q-learning [109], state-action-reward-state-action [110], deep q-network [111], Monte Carlo method [112]	DreamerV2 [113]
	Meta learning/Auto ML	Grid search [114], random search [114], Bayesian optimization [115]	Neural architecture search [116], Hyperband [117], Meta-Learning [118] (MAML [119], Reptile [120])

**Table 2:** Database-related information.

No.	Database	Origin of Data	Description	URL
1	Khazazna	computational	thermoplastic; mechanical, thermal, electrical properties	https://khazana.gatech.edu/dataset/ (accessed on 18 February 2020)
2	PolyInfo	empirical	thermoplastic; mechanical, optical, thermal, rheological properties	https://polymer.nims.go.jp/ (accessed on 22 January 2021)
3	Polymer property predictor and database	empirical	Flory–Huggins parameter, glass transition temperature, binary polymer solution cloud point	https://pppdb.uchicago.edu/ (accessed on 30 March 2016)
4	Material properties database	empirical/computational	thermoplastic, thermoset, rubber; mechanical, thermal, electrical properties	https://www.makeitfrom.com/ (accessed on 16 April 2020)
5	CROW polymer properties database	empirical/computational	thermoplastic, rubber, fiber; physical, thermal properties	https://polymerdatabase.com/ (accessed on 2 March 2019)
6	PI1M	computational	virtual polymers; physical, thermal, electrical properties	https://github.com/RUIMINMA1996/PI1M (accessed on 11 December 2020)
7	Dortmund database	computational	physical properties, phase equilibrium data	https://ddbst.com/ (accessed on 7 October 2020)
8	AI plus Polymers	empirical/computational	thermoset, thermoplastic; physical, mechanical, thermal, electrical properties	https://polymergenome.ecust.edu.cn/ (accessed on 23 March 2019)

## Data Availability

No new data were created or analyzed in this study.
